# Postoperative clinical outcomes and inflammatory markers after inguinal hernia repair using local, spinal, or general anesthesia: A randomized controlled trial

**DOI:** 10.1371/journal.pone.0242925

**Published:** 2020-11-30

**Authors:** Mingkwan Wongyingsinn, Pasawang Kohmongkoludom, Atthaphorn Trakarnsanga, Navin Horthongkham

**Affiliations:** 1 Department of Anesthesiology, Faculty of Medicine Siriraj Hospital, Mahidol University, Bangkok, Thailand; 2 Division of General Surgery, Department of Surgery, Minimally Invasive Surgery Unit, Faculty of Medicine Siriraj Hospital, Mahidol University, Bangkok, Thailand; 3 Department of Microbiology, Faculty of Medicine Siriraj Hospital, Mahidol University, Bangkok, Thailand; Cleveland Clinic, UNITED STATES

## Abstract

**Background:**

No consensus has yet been reached regarding the best anesthetic technique for inguinal hernia repair. This study aimed to compare postoperative clinical outcomes and inflammatory markers among patients who were anesthetized using local, spinal, or general anesthesia for inguinal hernia repair.

**Methods:**

This randomized controlled trial included patients scheduled to undergo elective unilateral inguinal hernioplasty at Siriraj Hospital during November 2014 to September 2015 study period. Patients were randomly assigned to the local (LA), spinal (SA), or general (GA) anesthesia groups. Primary outcomes were postoperative pain at rest and on mobilization at 8 and 24 hours after surgery.

**Results:**

Fifty-four patients were included, with 18 patients randomly assigned to each group. Patient demographic and clinical characteristics were similar among groups. There were no significant differences among groups for postoperative pain at rest or on mobilization at 8 and 24 hours after surgery. No significant differences were observed for interleukin-1β, interleukin-6, and interleukin-10 at any time points in any groups. Patients with local anesthesia was associated with less time spent in anesthesia (*p* = 0.010) and surgery (*p* = 0.009), lower intraoperative cost (*p* = 0.003) and total cost in hospital (*p* = 0.036); however, patient satisfaction in the local anesthesia group (94/100) was statistically significantly lower than the spinal and general anesthesia groups (100/100) (*p* = 0.010).

**Conclusions:**

No statistically significant difference was observed among groups for postoperative pain scores, duration of hospital stays, complications, or change in inflammatory markers. However, time spent in anesthesia and surgery, the intraoperative cost and total cost for hernia repair, and patient satisfaction were significantly lower in the local anesthesia group than in the other two groups.

## Background

Inguinal hernia repair is a commonly performed surgical procedure, and is one type of ambulatory surgery. Any techniques that deliver better analgesia, earlier ambulation, and shorter hospitalization are considered to be important. The choice of anesthetic technique has a major impact on how the patient responds during the postoperative period. Local anesthesia, spinal anesthesia, and general anesthesia are commonly used for this operation. While spinal anesthesia has been shown to decrease postoperative pain after inguinal herniorrhaphy when compared with general anesthesia; local anesthesia has been shown to reduce hospital time, lower the cost of treatment, and has no or fewer side effects compared with spinal and general anesthesia [1–6]. Although local anesthesia has been shown to be superior to the other two anesthetic methods in some clinical aspects, some studies have reported lower patient satisfaction, and a higher risk of recurrence when using local anesthesia [7–9]. No consensus has yet been reached regarding the best anesthetic technique for inguinal hernia repair.

Surgical manipulation associates with degree of inflammatory response, and leads to various postoperative outcomes. There is significant evidence showing that certain cytokines are directly involved in the activation of nociceptive sensory neurons [10]. Two studies reported a relationship between inflammatory response and both the number of postoperative days before recovery and length of hospital stay [11, 12]. Interventions and equipment that affect acute inflammatory response are considered to be important factors that can be manipulated to affect surgical outcomes. Examples of these factors include surgical manipulation and prosthetic mesh materials, both of which induce physiological changes and acute inflammatory response. Many studies have investigated changes in inflammatory serum markers according to mesh implantation material and/or surgical technique [13–17]. A 2001 study by Carli and Mayo proposed an outcome measurement strategy that took into consideration the link between biology and outcome [18]. Local anesthetics have a wide range of anti-inflammatory actions via their effects on cells of the immune system; however, the effect of local anesthetics on inflammatory response, and the relationship between the inflammatory process and clinical outcomes in inguinal hernia repair have not yet been established [19–28]. The aim of this study was to compare postoperative clinical outcomes and inflammatory markers among patients who were anesthetized using local, spinal, or general anesthesia for inguinal hernia repair.

## Material and methods

### Patient selection

The research protocol for this randomized controlled trial was approved by the Human Research Protection Unit of the Faculty of Medicine, Siriraj Hospital, Mahidol University, Thailand (*Si*157/2013) and was registered with ClinicalTrials.gov (NCT01845376). We included patients scheduled to undergo elective unilateral inguinal hernia repair at the Minimally Invasive Surgery Center, Faculty of Medicine Siriraj Hospital, Mahidol University (Bangkok, Thailand) during November 2014 to September 2015 study period. Patients aged older than 18 years with an ASA physical status classification of I, II, or III were recruited at the outpatient surgical department. Written informed consent was obtained from all participating patients. Patients having any one or more of the following were excluded: allergy to any medication used the study; femoral hernia; recurrent hernia; bilateral hernia; bleeding abnormalities; severe hepatic, renal, or cardiovascular disease; chronic use of opioid; history of using steroidal or nonsteroidal anti-inflammatory drugs within the past 6 months; inability to communicate; and/or, inability to understand the purpose of the study.

### Anesthesia and surgical procedure

All patients were routinely admitted one day prior to operation and patients received no premedication. Patients were randomly allocated on the morning of the operation to receive one of three anesthetic techniques [local anesthesia (LA); spinal anesthesia (SA); or, general anesthesia (GA)] for their inguinal hernia repair. Randomization software was used to allocate patients, and the group assignment numbers were sealed in individual brown envelopes. All included patients underwent standardized inguinal hernia repair by surgeons who agreed to follow a precise protocol using Lichtenstein technique, as described by Amid [29].

Patients in the LA group received local anesthesia according to a simple six-step infiltration technique as the protocol described by Amid, *et al*. with 0.5% bupivacaine plus 2% lidocaine with adrenaline (1:200,000) by surgeons that were instructed to perform this local anesthetic technique in a standardized manner [30]. Patients in the SA group were positioned in the lateral position and injected with 0.5% heavy bupivacaine 15 mg into the L3-4 intervertebral space using a Whitacre 25 G needle. Sensory block (dermatomes T4 and below) to cold and pinprick was tested before starting the operation. An incremental dose containing 1 mg of midazolam and 25 mcg of fentanyl was given intravenously if required by patients in the LA and SA groups. Patients in the GA group were induced with propofol 2 mg/kg and fentanyl 1.5 mcg/kg. They were allowed to breathe spontaneously with sevoflurane 2% to 2.5% in a mixture of 60% oxygen through a laryngeal mask. End-tidal concentration of sevoflurane was adjusted to maintain end-tidal sevoflurane at 1 MAC. Supplemental doses of 25 mcg of fentanyl were administered if intraoperative heart rate and blood pressure were greater than 20% of baseline.

For postoperative pain control, patients in the LA and GA groups were infiltrated with 0.5% bupivacaine 10 ml into the surgical wounds after the operation was concluded. Patients in all 3 groups received oral acetaminophen 500–1000 mg. every 6 hours and Etoricoxib (Arcoxia^®^) (Merck Sharp & Dohme, Kenilworth, NJ, USA) 60–90 mg daily (unless contraindicated) for the duration of their hospital stay. Intravenous morphine 1–2 mg was provided every 4 hours as a breakthrough pain medication. Postoperative anesthetic nurses and ward nurses followed their respective routine care pathways. Patients were discharged with no restrictions on activities, and they were encouraged to resume work and normal daily activities as soon as possible.

### Blood sample

Blood samples were collected in an anticoagulant tube on a preoperative day and at 8 and 24 hours after surgery to test for serum levels of interleukin-1 beta (IL-1 beta), interleukin-6 (IL-6), and interleukin-10 (IL-10). Blood samples were centrifuged for 15 min at 1,000 *x g*, and serum was stored at -80°C until used for cytokine testing. Serum IL-1 beta, IL-6, and IL-10 were assessed using a LEGEND MAX^™^ Human Interleukin ELISA Kit (Biolegend, Inc., San Diego, CA, USA). Briefly, the quantitative sandwich enzyme immunoassay technique used monoclonal antibodies specific to IL-1 beta, IL-6, and IL-10. Serum concentrations were calculated using regression analysis with standard curves and expressed as picograms per milliliter (pg/ml). All samples were measured in duplicate, with averages used for statistical analysis. The minimum detectable concentrations of IL-1 beta, IL-6, and IL-10 were 0.5 pg/ml, 1.6 pg/ml, and 2 pg/ml, respectively.

### Data collection and outcome measures

All germane perioperative data were collected from patient charts and input into a case record form by one of the authors. The following data were collected: demographic characteristics, diagnosis, duration of anesthesia and surgery, conversion to other anesthetic techniques or other operations, quality of pain relief, postoperative use of analgesic medication, intraoperative and postoperative complications, incidence of postoperative nausea and vomiting (PONV), length of postoperative hospital stay, acute inflammatory markers, patient satisfaction, incidence of complications, and readmission rate during 30 days after the operation. Duration of surgery was defined as the time from surgical incision to surgical end time. Duration of anesthesia was defined as the time when the anesthesiologist first made contact with the patient in the operating room to administer general anesthesia or regional or sedatives until the time when the patient was transferred to the postanesthesia care unit excluding surgical time. Complications were defined as bleeding or hematoma necessitating reoperation or compression bandage, urinary retention that required catheterization, and fever >38°C that required medication treatment.

Primary outcome was postoperative pain on mobilization at 24 hours after surgery, as measured by verbal rating scale (VRS) that ranged from 0 (no pain) to 10 (worst pain). Additionally, postoperative pain on mobilization were recorded at 8 hours and pain at rest were recorded at 2, 8 and 24 hours after surgery. Secondary outcome measures were acute inflammatory markers. Intermediate outcomes included duration of anesthesia and surgery, conversion to other anesthetic techniques, postoperative use of analgesics and amount of analgesic medication, incidence of nausea and vomiting, length of postoperative hospital stay in hours, patient satisfaction measured by verbal rating scale (VRS) that ranged from 0 (worst) to 100 (best), incidence of complications, readmission rate during 30 days after the operation, cost of intraoperative period and total cost for inguinal hernia repair.

### Sample size calculation and statistical analysis

The primary outcome was postoperative pain on mobilization at 24 hours after the operation. The sample size was based on data from two previous studies [2, 4]. Using nQuery Advisor version 7.0 (Statistical Solutions, Cork, Ireland), a balanced analysis of variance (ANOVA) test was performed to obtain a type I error of 0.05 and a power of 80%. The calculated sample size per group was 15 patients, which was increased to 18 patients per group to compensate for a dropout rate that was estimated not to exceed 20%.

Statistical analysis was performed using SPSS Statistics version 18 (SPSS, Inc., Chicago, IL, USA) (S1 File). Categorical variables were analyzed using chi-square test or Fisher’s exact test with compare column proportions and adjust p-values (Bonferroni method), and continuous variables were analyzed using either ANOVA with Bonferroni post hoc test for normally distributed data or Kruskall-Wallis test for non-normally distributed data. Categorical variables are presented as number and percentage, and continuous variables are presented as mean ± standard deviation or median and range (min–max). All statistical tests were two-tailed, and a *p*-value of less than 0.05 was regarded as being statistically significant.

## Results

**Fig 1:**
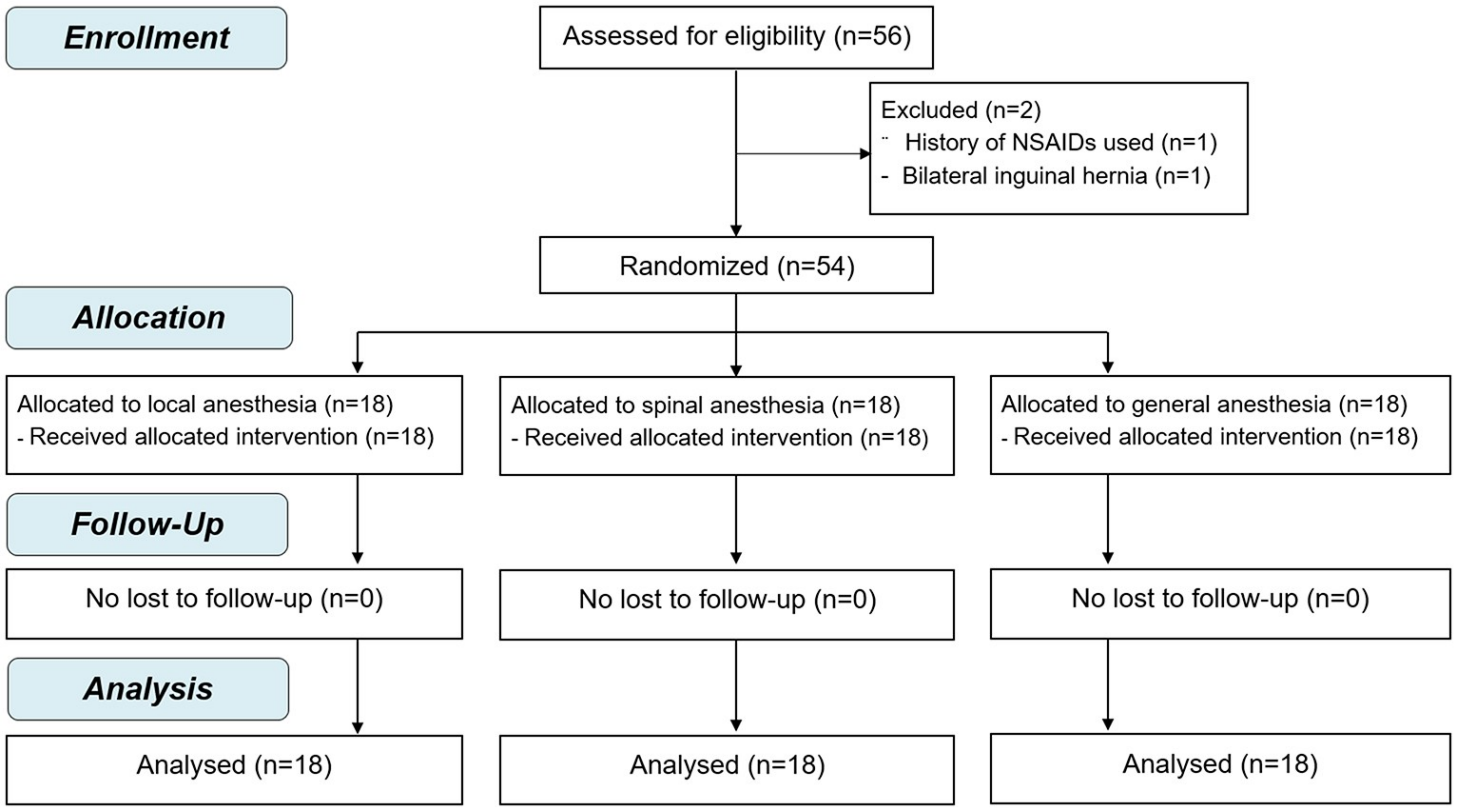
CONSORT flow diagram.

Of the 56 patients that were eligible for enrollment, 54 patients were included. Two patients were excluded, as follows: recent history of NSAID use (1 patient) and bilateral inguinal hernia (1 patient). Eighteen patients were randomly assigned to each of the LA, SA, and GA groups. After randomization, there were no conversions to other anesthetic techniques or other operations, and there was no loss to follow-up. The CONSORT flow diagram is shown in Fig 1.

Patient demographic and clinical characteristics, including age, gender, and body mass index, were similar among groups. American Society of Anesthesiologists (ASA) physical status, comorbidities, and type and side inguinal hernia were also similar among groups (Table 1). Intraoperative data, including amount of intravenous fluid administration, estimated blood loss, duration of surgery and anesthesia, and number of patients who had episodes of hypotension or bradycardia, are presented in Table 2. Duration of anesthesia was longest in the SA group (ANOVA; *p* = 0.010) and the duration in the SA group was also longer than the LA group significantly (*p* = 0.014) with posthoc analysis. However, patients in the GA group spent operative time and total time in operating theater for longest duration (*p* = 0.009 and *p* = 0.005). The operative time and total time in operating theater in the GA group were longer than the LA group significantly with posthoc analysis (*p* = 0.008 and *p* = 0.004, respectively). The incidences of intraoperative bradycardia were similar among groups (*p* = 0.214). In contrast, the incidence of hypotension in the GA group was statistically higher than other two groups (*p*<0.001); meanwhile, the incidence of hypotension in the RA group was not different significantly from the LA group with adjusted Bonferroni p-values (*p*>0.05). No patient showed signs of local anesthetic toxicity during the perioperative period.

**Table 1:** Demographic and clinical characteristics of patients by study group.

Variables	LA group	SA group	GA group
	(n = 18)	(n = 18)	(n = 18)
Age (years)	66.1 ± 15.1	64.9 ± 10.3	67.7 ± 15.1
Gender: male	18 (100%)	17 (94.4%)	18 (100%)
Weight (kg)	61.4 ± 7.3	61.3 ± 6.8	66.2 ± 6.5
Body mass index (kg/m^2^)	22.2 ± 1.8	22.7 ± 1.9	23.1 ± 2.6
ASA physical status			
I	4 (22.2%)	3 (16.7%)	3 (16.7%)
II	11 (64.1%)	7 (38.9%)	8 (44.4%)
III	3 (16.7%)	8 (44.4%)	7 (38.9%)
Comorbidity			
Hypertension	9 (50%)	9 (50%)	8 (44.4%)
Diabetes mellitus	3 (16.7%)	2 (11.1%)	3 (16.7%)
Dyslipidemia	5 (27.8%)	3 (16.7%)	4 (22.2%)
Chronic kidney disease/renal insufficiency	2 (11.1%)	4 (22.2%)	3 (16.7%)
Gout	2 (11.1%)	4 (22.2%)	1 (5.6%)
Benign prostatic hyperplasia	2 (11.1%)	2 (11.1%)	4 (22.2%)
Cerebrovascular accident	1 (5.6%)	2 (11.1%)	2 (11.1%)
Coronary artery disease	0	1 (5.6%)	3 (16.7%)
Chronic obstructive pulmonary disease	1 (5.6%)	0	1 (5.6%)
Obstructive sleep apnea	1 (5.6%)	1 (5.6%)	0
Cirrhosis	0	1 (5.6%)	1 (5.6%)
Asthma	1 (5.6%)	0	0
Type of hernia			
Direct	13 (72.2%)	16 (88.9%)	18 (100%)
Indirect	5 (27.8%)	2 (11.1%)	0
Side of hernia:			
Right	9 (50%)	6 (33.3%)	9 (50%)
Left	9 (50%)	12 (66.7%)	9 (50%)

Data presented as mean ± standard deviation, or number and (percentage).

Abbreviations: LA, local anesthesia; SA, spinal anesthesia; GA, general anesthesia; ASA, American Society of Anesthesiologists.

**Table 2:** Intraoperative data by study group.

Variables	LA group	SA group	GA group	*p-*value
	(n = 18)	(n = 18)	(n = 18)	
Amount of IV fluid replacement (mL)	200 (100–700)	350 (100–1,100)	500 (100–1,000)	0.038
Estimated blood loss (mL)	5 (1–20)	7.5 (1–30)	7.5 (0–40)	0.339
Duration of anesthesia (min)	16.8 ± 7.2*	25.5 ± 9.4*	23.9 ± 9.5	0.010
Duration of surgery (min)	63.7 ± 15.6*	71.9 ± 17.9	86.6 ± 28.9*	0.009
Total duration in operating theater (min)	82.8 ± 15.8*	98.9 ± 20.8	109.4 ± 31.0*	0.005
Number of patients having hypotension	0 (0%)^†^	4 (22.2%)^††^	12 (66.7%)^†^^,^ ^††^	<0.001
Number of patients having bradycardia	0 (0%)	2 (11.1%)	3 (16.7%)	0.214

Data presented as median (min–max), mean ± standard deviation, or number and (percentage).

Abbreviations: LA, local anesthesia; SA, spinal anesthesia; GA, general anesthesia.

A *p*-value<0.05 indicates statistical significance.

*Post-hoc test with Bonferroni correction showed significant difference between groups.

^†,††^ Comparison proportions with adjusted Bonferroni p-values showed significant difference between groups.

Hypotension was defined as a decrease in blood pressure below 20% of baseline for more than 10 minutes.

Bradycardia was defined as a heart rate less than 60/min and/or a rapidly falling heart rate.

**Fig 2:**
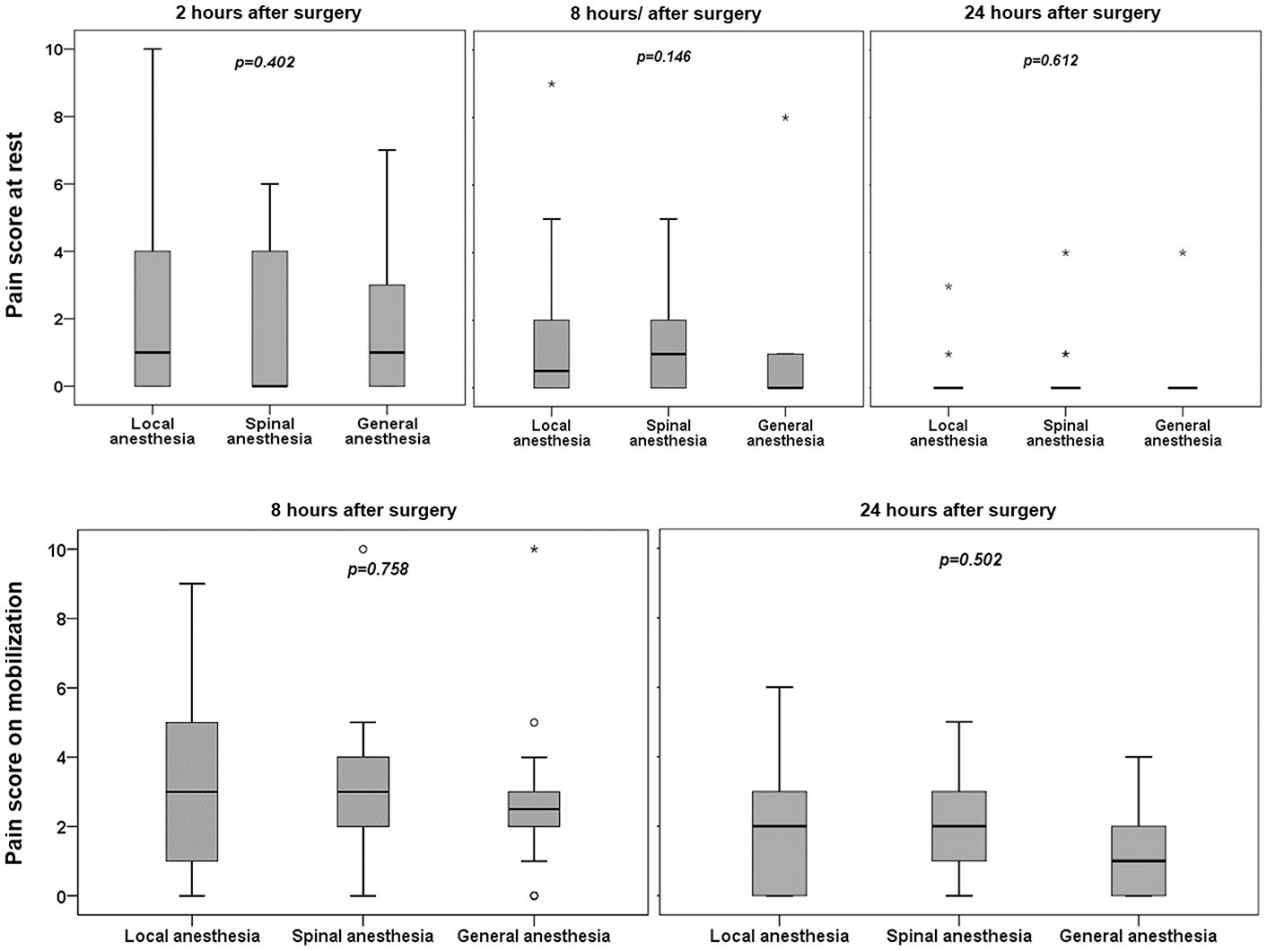
Postoperative pain score at rest and on mobilization at 8 and 24 hours after surgery. ° = Outliers = Subjects with values between P75 + 1.5 IQR and P75 + 3 IQR. * = Extremes = Subjects with values higher than P75 + 3 IQR. The Kruskal-Wallis test was used to compare mean rank among comparison groups.

There were no significant differences among groups for postoperative pain at rest or on mobilization at 8 and 24 hours after surgery (Fig 2). The number of patients requiring rescue morphine and the total amount of morphine administered in the postoperative care unit during the first 2 hours after surgery were highest in the general anesthesia group (*p*<0.001), but no significant difference was observed among three groups at the 24-hour time point in both number of patients requiring morphine and the total amount of morphine administered (*p* = 0.216 and *p* = 0.064, respectively). There were no differences in the use of oral analgesics among the groups (Table 3).

**Table 3:** Postoperative data by study group.

Variables	LA group	SA group	GA group	*p-*value
	(n = 18)	(n = 18)	(n = 18)	
Number of patients receiving				
Acetaminophen	18 (100%)	18 (100%)	18 (100%)	1.000
Etoricoxib (Arcoxia^®^)	11 (61.1%)	8 (44.4%)	11 (61.1%)	0.651
In PACU				
Number of patients requiring morphine	3 (16.7%)^†^	2 (11.1%)^††^	12 (66.7%)^†^^,^ ^††^	<0.001
Total amount of morphine administered (mg)	0 (0–0.8)*	0 (0–2)**	1 (0–5)*^,^ **	<0.001
In the first 24 hours				
Number of patients requiring morphine	10 (55.6%)	9 (50%)	14 (77.8%)	0.216
Total amount of morphine administered (mg)	0.5 (0–6)	0.3 (0–6)	1.5 (0–5)	0.064
Discharge from hospital				0.551
Postoperative day 1	16 (88.9%)	17 (94.4%)	16 (88.9%)	
Postoperative day 2	0	1 (5.6%)	1 (5.6%)	
Postoperative day 3	2 (11.1%)	0	1 (5.6%)	
Complications	3 (17%)	3 (17%)	6 (33%)	0.542
Revisiting hospital	0 (0%)	0 (0%)	1 (5.6%)	1.000
Satisfaction score	94 (50–100)*	100 (90–100)*	100 (70–100)	0.010

Data presented as median (min–max), or number and (percentage).

Abbreviations: LA, local anesthesia; SA, spinal anesthesia; GA, general anesthesia; PACU, post-anesthesia care unit.

A *p*-value<0.05 indicates statistical significance.

^†,††^ Comparison proportions with adjusted Bonferroni p-values showed significant difference between groups.

*, **Pairwise comparison with Kruskal-Wallis ANOVA showed significant difference between groups.

Pro-inflammatory cytokines nterleukin-1β, interleukin-6, and anti-inflammatory cytokine interleukin 10 (IL-10) were compared between preoperative baseline and 8 and 24 hours after surgery to detect change in serum level. No significant differences were observed at any time points for any of the 3 anesthetic groups (Table 4).

**Table 4:** Inflammatory markers at preoperative baseline, 8 and 24 hours after surgery.

Variables	LA group	SA group	GA group	*p-*value
	(n = 18)	(n = 18)	(n = 18)	
IL-10 level (pg/ml)				
Preoperative baseline	0 (0–4.2)	0 (0–17.5)	0 (0–3.3)	0.545
8 hours after surgery	4.7 (0–7.2)	5.1 (0–19.6)	3.7 (0–10.5)	0.911
24 hours after surgery	5.4 (0–13.8)	5.7 (0–19.2)	5.2 (0–10.9)	0.628
IL-6 level (pg/ml)				
Preoperative baseline	0.9 (0–38.2)	2.3 (0–121.1)	0 (0–8.3)	0.318
8 hours after surgery	30.4 (6.8–78.3)	31.6 (10.4–169.8)	29.8 (9.4–102.3)	0.972
24 hours after surgery	16.6 (2.3–126.8)	16.1 (2.5–149.3)	15.6 (2.6–71.4)	0.879
IL-1β level (pg/ml)				
Preoperative baseline	0	0	0	
8 hours after surgery	0.5 (0.5–2.6)	0.5 (0.5–3.1)	0.5 (0.5–4.0)	0.761
24 hours after surgery	0.5 (0.5–3.1)	0.5 (0.5–2.9)	0.5 (0.5–4.2)	0.493

Data presented as median (min–max).

Abbreviations: LA, local anesthesia; SA, spinal anesthesia; GA, general anesthesia.

*p*-value for Kruskal-Wallis test.

The postoperative complications were not statistically different among three anesthetic techniques. Four patients in the GA group (one patient had postoperative shivering in the post-anesthesia care unit, one patient had numbness around incision, one patient had postoperative neuralgia and another patient had redness in the area of the wound), two patients in the SA group had urinary retention, two patients in the LA group (one patient had postoperative nausea and vomiting, and another patient had small wound hematoma). All eight patients had supportive treatment and the total of 49 patients (91%) were discharged from the hospital on postoperative day 1. One patient in the GA group had scrotal ecchymosis, and another patient in the SA group had numbness at the medial thigh of one leg; both patients had supportive treatment and were discharged on postoperative day 2. Three patients were discharged from the hospital on postoperative day 3 for the following reasons: postoperative fever (one patient in the GA group), large wound hematoma (one patient in the LA group), and social reason (one patient in the LA group). One patient in the GA group revisited the hospital at two weeks after discharge and was diagnosed with an infected mesh. That patient underwent local debridement with no readmission, and then went on to make a full recovery.

**Fig 3:**
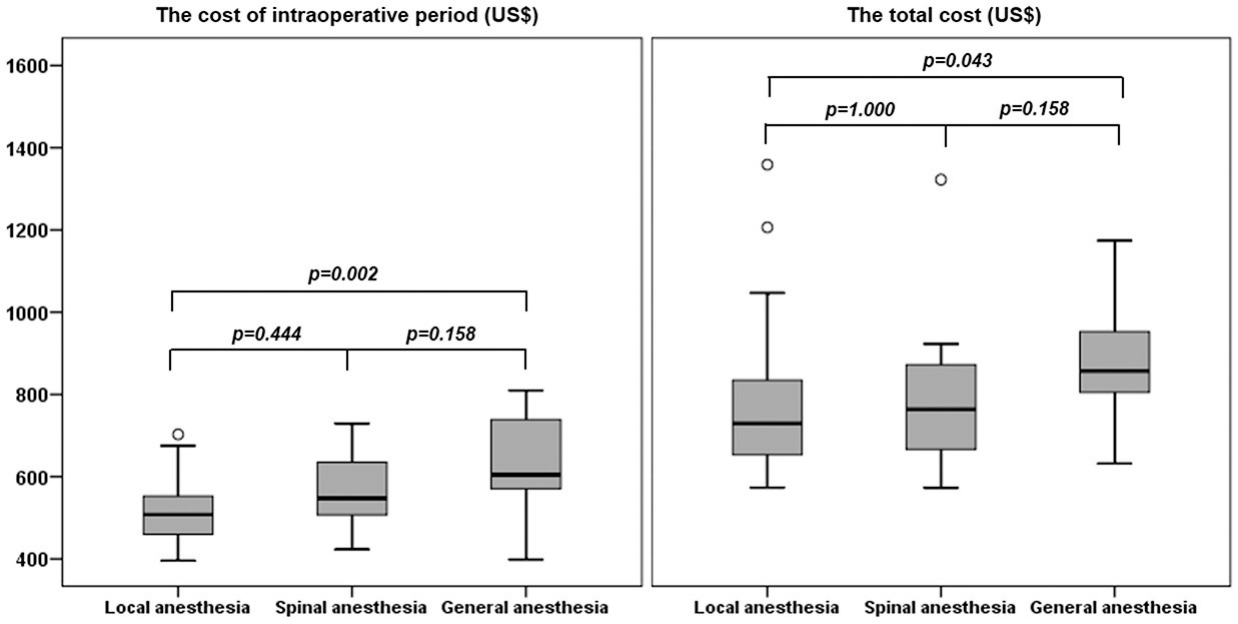
The intraoperative costs and total costs for inguinal hernia repair using three anesthetic techniques. ° = Outliers = Subjects with values between P75 + 1.5 IQR and P75 + 3 IQR. * = Extremes = Subjects with values higher than P75 + 3 IQR. The Kruskal-Wallis test was used to compare mean rank among comparison groups.

The median patient satisfaction score was lowest in the LA group (94/100), while the other two groups had a median score of 100/100. The average intraoperative costs were different among three anesthetic techniques (*p* = 0.003), and the total costs of inguinal hernia repair associated with using different anesthetic techniques were also significantly different (*p* = 0.036). The local anesthesia had a lower intraoperative cost (US$507.81) compared with the general anesthesia (US$604.71, *p* = 0.002), while the intraoperative cost of the local anesthesia was not different with the spinal anesthesia (US$547.39). The total cost of inguinal hernia repair was also lowest with the local anesthesia (US$729.44) and increased with the spinal anesthesia (US$763.88) and the general anesthesia (US$856.79) respectively (Fig 3).

## Discussion

In this study, postoperative pain scores at 2, 8 and 24 hours were not significantly different among the three anesthetic techniques. However, the numbers of patients required rescue morphine and the morphine consumption in the post-anesthetic care unit were highest in the GA group but there was no significant difference among groups at the 24-hour time point in both number of patients receiving rescue morphine and the total amount of rescue morphine administered.

Data on postoperative pain regarding types of anesthesia showed a consistent pattern of reduced pain with the local anesthesia technique [2, 31]. However, postoperative pain scores in this study were not different among the three anesthetic techniques and were similar to postoperative pain score at 24 hours reported in a 2003 study, with no major differences in patient recovery between local anesthesia and general anesthesia for hernia repair [7].

During the first two hours in the post-anesthetic care unit, the number of patients requiring rescue pain medication under local anesthesia was less than patients having operation under general anesthesia which was similar to a 2003 study [7]. While O’Dwyer, *et al*. reported 66% of patients with local anesthesia required rescue analgesics in the recovery room, our study reported only 17% in this technique [7]. The reasons to explain this different percentage may come from various surgical and local anesthetic infiltration techniques by various surgeons, although the standardized inguinal hernia repair and infiltration technique were recommended to use in both studies [29, 30]. Regarding postoperative opioid consumption in this study, patients with local anesthesia also consumed lower amount of morphine in the recovery room, which was similar to the results of a previous study [7, 31]. However, the cumulative morphine consumption at 24 hours postoperatively was not significantly different among groups and was similar to that reported by O’Dwyer, *et al*. study [7].

Recent studies showed the benefit of local anesthesia in patients undergoing open inguinal hernia repair in terms of shorter anesthesia and operative times compared with spinal or general anesthesia, which were consistent with this study about the anesthetic time, operative time, and total time in operating theater [31–33]. While local anesthesia need time to infiltrate local anesthetics step by step; general and spinal anesthesia needs more time to perform informative procedures [30]. The more time spent in operating theater caused more intraoperative expenses in spinal and general anesthesia, respectively.

Moreover, performing operation under local anesthesia reduced need for postoperative hospital admission, reduced postoperative morbidity, and improved short-term and long-term postoperative quality of life, when compared with general anesthesia [3, 31–33]. The data was persistent in this study with the high incidences of intraoperative hypotension and bradycardia in the GA group, while no patients in the local anesthesia group having either of these complications. The postoperative urinary retention occurred in two patients with spinal anesthesia while no patient under local or general anesthesia experienced this complication. Unfortunately, our study found no difference in hospital length of stay among groups as same as contrasted with previous study reported the shortest duration in the local anesthesia group [31, 33]. Our study reported the lowest level of patient satisfaction among those who received local anesthesia which is similar to a previous study [7].

This study has some mentionable limitations. First, although the Lichtenstein technique as described by Amid was recommended for this study, nuanced differences and variations in surgical technique still exist among surgeons. Second, this study measured interleukin levels at only 8 and 24 hours after surgery, which may not be frequent enough to detect changes in inflammatory markers. A previous study reported that interleukin-6 concentration increases within 30–60 minutes after the start of surgery and that the change in concentration becomes significant after 2–4 hours [34]. That same author went on to say that the change in concentration reaches its maximal level at about 24 hours and remains elevated for 48–72 hours after surgery. Accordingly, the study of change in blood serum interleukin levels might be more telling if continued to 48 and 72 hours after surgery. Moreover, inflammatory markers should be monitored at shorter intervals to facilitate the identification of short duration changes in inflammatory markers. Third, the interleukin level in this study, especially interleukin-1β, may be lower than the minimum detectable level that our lab could report. As such, a more sensitive of interleukin ELISA test may be required.

## Conclusion

No statistically significant difference was observed among the 3 anesthesia groups for postoperative pain scores, duration of hospital stays, complications, or change in inflammatory markers. Patients with local anesthesia was associated with less time spent in anesthesia and surgery, lower intraoperative cost and total cost in hospital; however, patient satisfaction group was statistically significantly lower than the spinal and general anesthesia groups. Therefore, surgeons and anesthesiologists should involve the patient in the anesthetic method decision by disclosing the benefits and drawbacks of each anesthetic method.

## Supporting information

S1 File Raw data of postoperative outcomes and inflammatory markers after inguinal hernia repair. (XLSX)

S1 Checklist CONSORT 2010 checklist of information to include when reporting a randomised trial. (DOC)

S1 Data (PDF)

## References

[1] Job CA, Fernandez MA, Dorph DJ, Betcher AM. Inguinal hernia repair. Comparison of local, epidural, and general anesthesia. N Y State J Med. 1979;79(11):1730–3. 290883

[2] Tverskoy M, Cozacov C, Ayache M, Bradley EL Jr., Kissin I. Postoperative pain after inguinal herniorrhaphy with different types of anesthesia. Anesth Analg. 1990;70(1):29–35. 10.1213/00000539-199001000-00006 2297102

[3] Callesen T, Bech K, Kehlet H. One-thousand consecutive inguinal hernia repairs under unmonitored local anesthesia. Anesth Analg. 2001;93(6):1373–6, table of contents. 10.1097/00000539-200112000-00004 11726409

[4] Sungurtekin H, Sungurtekin U, Erdem E. Local anesthesia and midazolam versus spinal anesthesia in ambulatory pilonidal surgery. J Clin Anesth. 2003;15(3):201–5. 10.1016/s0952-8180(03)00032-1 12770656

[5] Kehlet H, Aasvang E. Groin hernia repair: anesthesia. World J Surg. 2005;29(8):1058–61. 10.1007/s00268-005-7969-8 15981039

[6] Chang FC, Farha GJ. Inguinal herniorrhaphy under local anesthesia. A prospective study of 100 consecutive patients with emphasis of perioperative morbidity and patient acceptance. Arch Surg. 1977;112(9):1069–71. 10.1001/archsurg.1977.01370090051009 901173

[7] O’Dwyer PJ, Serpell MG, Millar K, Paterson C, Young D, Hair A, et al Local or general anesthesia for open hernia repair: a randomized trial. Ann Surg. 2003;237(4):574–9. 10.1097/01.SLA.0000059992.76731.64 12677155 PMC1514474

[8] Kingsnorth AN, Britton BJ, Morris PJ. Recurrent inguinal hernia after local anaesthetic repair. Br J Surg. 1981;68(4):273–5. 10.1002/bjs.1800680416 7225743

[9] Nordin P, Haapaniemi S, van der Linden W, Nilsson E. Choice of anesthesia and risk of reoperation for recurrence in groin hernia repair. Ann Surg. 2004;240(1):187–92. 10.1097/01.sla.0000130726.03886.93 15213635 PMC1356391

[10] Zhang JM, An J. Cytokines, inflammation, and pain. Int Anesthesiol Clin. 2007;45(2):27–37. 10.1097/AIA.0b013e318034194e 17426506 PMC2785020

[11] Cruickshank AM, Fraser WD, Burns HJ, Van Damme J, Shenkin A. Response of serum interleukin-6 in patients undergoing elective surgery of varying severity. Clin Sci (Lond). 1990;79(2):161–5. 10.1042/cs0790161 2167805

[12] Kehlet H. Surgical stress response: does endoscopic surgery confer an advantage? World J Surg. 1999;23(8):801–7. 10.1007/s002689900583 10415206

[13] Di Vita G, Milano S, Frazzetta M, Patti R, Palazzolo V, Barbera C, et al Tension-free hernia repair is associated with an increase in inflammatory response markers against the mesh. The American Journal of Surgery. 2000;180(3):203–7. 10.1016/s0002-9610(00)00445-1 11084130

[14] Hill AD, Banwell PE, Darzi A, Menzies-Gow N, Monson JR, Guillou PJ. Inflammatory markers following laparoscopic and open hernia repair. Surg Endosc. 1995;9(6):695–8. 10.1007/BF00187942 7482166

[15] Gürleyik E, Gürleyik G, Çetinkaya F, Ünalmiser S. The Inflammatory Response to Open Tension-free Inguinal Hernioplasty Versus Conventional Repairs. The American Journal of Surgery. 1998;175(3):179–82. 10.1016/s0002-9610(97)00293-6 9560115

[16] Goldenberg A, Matone J, Marcondes W, Herbella FA, Farah JF. Comparative study of inflammatory response and adhesions formation after fixation of different meshes for inguinal hernia repair in rabbits. Acta Cir Bras. 2005;20(5):347–52. 10.1590/s0102-86502005000500002 16186957

[17] Schwab R, Eissele S, Bruckner UB, Gebhard F, Becker HP. Systemic inflammatory response after endoscopic (TEP) vs Shouldice groin hernia repair. Hernia. 2004;8(3):226–32. 10.1007/s10029-004-0216-7 15042432

[18] Carli F, Mayo N. Measuring the outcome of surgical procedures: what are the challenges? Br J Anaesth. 2001;87(4):531–3. 10.1093/bja/87.4.531 11878719

[19] Cassuto J, Sinclair R, Bonderovic M. Anti-inflammatory properties of local anesthetics and their present and potential clinical implications. Acta Anaesthesiol Scand. 2006;50(3):265–82. 10.1111/j.1399-6576.2006.00936.x 16480459

[20] Giddon DB, Lindhe J. In vivo quantitation of local anesthetic suppression of leukocyte adherence. Am J Pathol. 1972;68(2):327–38. 5049429 PMC2032682

[21] MacGregor RR, Thorner RE, Wright DM. Lidocaine inhibits granulocyte adherence and prevents granulocyte delivery to inflammatory sites. Blood. 1980;56(2):203–9. 7397378

[22] Schmidt W, Schmidt H, Bauer H, Gebhard MM, Martin E. Influence of lidocaine on endotoxin-induced leukocyte-endothelial cell adhesion and macromolecular leakage in vivo. Anesthesiology. 1997;87(3):617–24. 10.1097/00000542-199709000-00023 9316968

[23] Azuma Y, Shinohara M, Wang PL, Suese Y, Yasuda H, Ohura K. Comparison of inhibitory effects of local anesthetics on immune functions of neutrophils. Int J Immunopharmacol. 2000;22(10):789–96. 10.1016/s0192-0561(00)00040-0 10963851

[24] Ohsaka A, Saionji K, Sato N, Igari J. Local anesthetic lidocaine inhibits the effect of granulocyte colony-stimulating factor on human neutrophil functions. Exp Hematol. 1994;22(5):460–6. 7513653

[25] Lan W, Harmon DC, Wang JH, Shorten GD, Redmond PH. Activated endothelial interleukin-1beta, -6, and -8 concentrations and intercellular adhesion molecule-1 expression are attenuated by lidocaine. Anesth Analg. 2005;100(2):409–12. 10.1213/01.ANE.0000142113.39092.87 15673867

[26] Hollmann MW, Gross A, Jelacin N, Durieux ME. Local anesthetic effects on priming and activation of human neutrophils. Anesthesiology. 2001;95(1):113–22. 10.1097/00000542-200107000-00021 11465548

[27] Mikawa K, Akamarsu H, Nishina K, Shiga M, Obara H, Niwa Y. Effects of ropivacaine on human neutrophil function: comparison with bupivacaine and lidocaine. Eur J Anaesthesiol. 2003;20(2):104–10. 10.1017/s026502150300019x 12622492

[28] Doran C, Yi X. The anti-inflammatory effect of local anesthetics. The Pain Clinic. 2013;19(5):207–13.

[29] Amid PK, Shulman AG, Lichtenstein IL. Open "tension-free" repair of inguinal hernias: the Lichtenstein technique. Eur J Surg. 1996;162(6):447–53. 8817221

[30] Amid PK, Shulman AG, Lichtenstein IL. Local anesthesia for inguinal hernia repair step-by-step procedure. Ann Surg. 1994;220(6):735–7. 10.1097/00000658-199412000-00004 7986138 PMC1234473

[31] Young DV. Comparison of local, spinal, and general anesthesia for inguinal herniorrhaphy. Am J Surg. 1987;153(6):560–3. 10.1016/0002-9610(87)90154-1 3592070

[32] Huntington CR, Wormer BA, Cox TC, Blair LJ, Lincourt AE, Augenstein VA, et al Local Anesthesia in Open Inguinal Hernia Repair Improves Postoperative Quality of Life Compared to General Anesthesia: A Prospective, International Study. Am Surg. 2015;81(7):704–9. 26140891

[33] Bhattacharya SD, Vaslef SN, Pappas TN, Scarborough JE. Locoregional versus general anesthesia for open inguinal herniorrhaphy: a National Surgical Quality Improvement Program analysis. Am Surg. 2012;78(7):798–802. 22748541

[34] Desborough JP. The stress response to trauma and surgery. Br J Anaesth. 2000;85(1):109–17. 10.1093/bja/85.1.109 10927999

