# Supplementary material for: Postoperative clinical outcomes and inflammatory markers after inguinal hernia repair using local, spinal, or general anesthesia: A randomized controlled trial
Source: PLoS One. 2020 Nov 30;15(11):e0242925. doi: 10.1371/journal.pone.0242925 (PMC7703886; doi:10.1371/journal.pone.0242925)
Supplement: S1 Data — (PDF) [file pone.0242925.s003.pdf]

# Postoperative Clinical Outcomes and Inflammatory Markers after Inguinal Hernia Repair with Local or Spinal or General Anesthesia: A Randomized Trial

**Mingkwan Wongyingsinn[1*], Pasawang Kohmongkoludom[1], Atthaphorn Trakarnsanga[2], Navin Horthongkham[3]**

[1] *Department of Anesthesiology, Faculty of Medicine Siriraj Hospital, Mahidol University, Bangkok, Thailand*

[2] *Division of General Surgery, Department of Surgery, Faculty of Medicine Siriraj Hospital, Mahidol University, Bangkok, Thailand*

[3] *Department of Microbiology, Faculty of Medicine Siriraj Hospital, Mahidol University, Bangkok, Thailand*

## Background

Inguinal hernia repair is one of the most common procedures and represents as an example of ambulatory surgery. Any techniques providing better analgesia, earlier ambulation, and shorter hospitalization are considered to be important factors. The choice of anesthetic technique is concerned to have a major impact on the postoperative period. Local anesthesia, spinal anesthesia and general anesthesia are commonly used for this operation. While spinal anesthesia has been shown to decrease postoperative pain after inguinal herniorrhaphy compared with general anesthesia; local anesthesia has been shown to provide a shorter hospital time, lower cost and no side effect compared with spinal and general anesthesia.(1-6) Although the local anesthesia seems to have superior results in many clinical aspects, some studies have reported less patients' satisfaction, lower benefits and more risk of recurrence rates with this technique.(7-9) Nowadays, there is still no consensus about the best anesthetic technique for inguinal hernia repair.

Surgical manipulation associates with degree of inflammatory response and leads to various postoperative outcomes. There is significant evidence showing that certain cytokines is directly involved in activating nociceptive sensory neurons.(10) One study in 2001 demonstrated that there was a relationship between the inflammatory response and the number of postoperative days before recovery and length of hospital stay.(11, 12) Interventions or equipment affecting the acute inflammatory response have been consider to be important factors to manipulate surgical outcomes including the prosthetic mesh and surgical manipulation which induce physiological changes and acute inflammatory response. Many studies have been published on the modifications of inflammatory serum markers with different mesh implantation or surgical technique.(13-17) Another study proposed that the measurement strategy considering the link between biology and outcome.(18) Local anesthetics is a medication having a wide range of anti-inflammatory actions through their effects on

cells of the immune system, however the effect of anesthetic technique with local anesthetics on the inflammatory response and the relation between the inflammatory process and clinical outcomes in inguinal hernia repair have not been done yet.[19-28]

This present randomized trial is designed to evaluate the postoperative pain, postoperative analgesic medication, length of hospital stay and the modifications of inflammatory mediators in patients undergoing inguinal hernia repair using local, spinal or general anesthesia.

## Methods

### *Patient selection*

After approval by Siriraj Institutional Review Board, a prospective randomized study in patients scheduled to undergo elective unilateral inguinal hernia repair at Siriraj Hospital will be undertaken. Patients, ASA I–III, greater than 18 years old will be approached in the outpatient surgical department and recruited in this study. Informed written consent will be obtained from each of them. Exclusion criteria are allergy to any medication used this study, femoral hernia, recurrent hernia, bilateral hernia, bleeding abnormalities, severe hepatic, renal or cardiovascular disease, chronic use of opioid, history of using steroidal or nonsteroidal anti-inflammatory drugs in the past 6 months, inability to communicate in Thai or to understand the purpose of the study.

### *Anesthesia and surgical procedure*

All patients will be admitted one day before operation as routine and receive no premedication. Patients will be randomly allocated in the morning of the operation to receive one of the three anesthetic techniques: local anesthesia (LA), spinal anesthesia (SA) or general anesthesia (GA) for their inguinal hernia repair. The randomization process will be done by the use of a computer-generated number sealed in a brown envelope. All the patients will undergo standardized inguinal hernia repairs by three surgeons, A Trakarnsanga; V Chinsawangtanakul; T Akaraviputh, who agree to follow a precise protocol using Lichtenstein technique as described by Amid.[29]

Patients in the LA group received local anesthesia according to a simple six-step infiltration technique as the protocol described by Amid, et al. with 0.5% bupivacaine plus 2% lidocaine with adrenaline (1:200,000) by surgeons.[30] Surgeons will be taught to do the local anesthetic technique in a standardized manner. In the SA group, patients will be positioned in the lateral position and a Whitacre 25 G needle will be inserted at L3-4 intervertebral space and then heavy bupivacaine 0.5% 15 mg will be injected. Sensory block (T4 and below dermatomes) to cold and pinprick will be tested before starting

operation. An incremental dose containing 1 mg of midazolam and 25 mcg of fentanyl will be intravenously given if patients in the LA and SA group require. In the GA group, patients will be induced with propofol 2 mg/kg and fentanyl 1.5 µg /kg. They are then allowed to breathe spontaneously with sevoflurane 2% to 2.5% in a mixture of 60% oxygen through a laryngeal mask. End-tidal concentration of sevoflurane will be adjusted to keep end-tidal sevoflurane 1MAC. Supplemental doses of 25 µg of fentanyl will be administered if intraoperative heart rate and blood pressure are greater than 20% of baseline.

For postoperative analgesia, all groups will receive infiltration 10 ml of 0.5% bupivacaine into the surgical wounds. They will also receive oral acetaminophen two tablets every 6 hours and Arcoxia® (etoricoxib) 60-90 mg daily unless contraindicated for the duration of their hospital stay. Intravenous morphine 1-2 mg will be provided every 4 hours as a breakthrough medication.

For hospital discharge, patients will be allowed to discharge home when they fulfill criteria: obtain and self-administer medications; perform self-care activities; eat an appropriate diet or otherwise manage nutritional needs; follow-up with designated providers.

***Blood samples***

A blood sample 7 mL will be taken at anticubital vein with aseptic technique, proper decontamination procedures and needle 22-gauge on the preoperative day, at 8 and 24 hours after completion of the surgery. Blood samples will be collected in tubes without anticoagulant to perform interleukin-1 beta (IL-1 beta), IL-6 and IL-10 assays. All blood samples will be centrifuged for 15 min at 1000*g.* Serum will be stored at −80 °C until performing assay for cytokines. The IL-1 beta, IL-6 and IL-10 will be assessed in the serum with LEGEND MAX Human Interleukin ELISA Kit (Biolegend, USA). Briefly, the quantitative sandwich enzyme immunoassay technique uses monoclonal antibodies specific for IL-1 beta or IL-6 or IL-10. Serum concentrations will be calculated by using regression analysis with standard curves and expressed as picograms per milliliter (pg /ml). All samples will be measured in duplicate, with averages used in the statistical analyses. The minimum detectable concentrations IL-1 beta, IL-6 and IL-10 are 0.5 pg /ml, 1.6 pg /ml and 2 pg /ml, respectively.

**Data collection**

All the perioperative data will be collected from patients' chart and filled in case record form by one of the authors. The ward nurses round and the postoperative anesthetic nurses round will follow the care pathway as routine. Patients have no

restrictions on activities, and they are encouraged to resume work and normal daily activities as soon as possible. Patients are discharged in accordance with routine at our respective hospital.

The following data will be collected: demographic characteristics, diagnosis, duration of anesthesia and surgery, conversion to other anesthetic techniques or other operations, quality of pain relief, postoperative use of analgesics and amount of analgesic medication, intraoperative and postoperative complications, incidence of postoperative nausea and vomiting (PONV), length of postoperative hospital stay, acute inflammatory markers, patient satisfaction, incidence of complications and readmission rate during 30 days after the operation. Complications are defined as bleeding or hematoma necessitating reoperation or compression bandage, urinary retention that requires catheterization, fever > 38°Celsius requiring medication treatment.

**Outcome measures**

Primary outcome was postoperative pain on mobilization at 24 hours after surgery, as measured by verbal rating scale (VRS) that ranged from 0 (no pain) to 10 (worst pain). Additionally, postoperative pain on mobilization were recorded at 8 hours and pain at rest were recorded at 2, 8 and 24 hours after surgery. Secondary outcome measures were acute inflammatory markers. Intermediate outcomes included duration of anesthesia and surgery, conversion to other anesthetic techniques, postoperative use of analgesics and amount of analgesic medication, incidence of nausea and vomiting, length of postoperative hospital stay in hours, patient satisfaction measured by verbal rating scale (VRS) that ranged from 0 (worst) to 100 (best), incidence of complications, readmission rate during 30 days after the operation, cost of intraoperative period and total cost for inguinal hernia repair.

**Sample size calculation**

The primary outcome was postoperative pain on mobilization at 24 hours after the operation. The primary outcome was postoperative pain on mobilization at 24 hours after the operation. The sample size was based on data from two previous studies.[2, 4] Using nQuery Advisor version 7.0 (Statistical Solutions, Cork, Ireland), a balanced analysis of variance (ANOVA) test was performed to obtain a type I error of 0.05 and a power of 80%. The calculated sample size per group was 15 patients, which was increased to 18 patients per group to compensate for a dropout rate that was estimated not to exceed 20%.

**Statistical analysis**

Statistical analysis was performed using SPSS Statistics version 18 (SPSS, Inc., Chicago, IL, USA). Categorical variables were analyzed using chi-square test or Fisher's exact test with compare column proportions and adjust p-values (Bonferroni method), and continuous variables were analyzed using either ANOVA with Bonferroni post hoc test for normally distributed data or Kruskall-Wallis test for non-normally distributed data. Categorical variables are presented as number and percentage, and continuous variables are presented as mean ± standard deviation or median and range (min – max). All statistical tests were two-tailed, and a p-value of less than 0.05 was regarded as being statistically significant.

**References**

1. Job CA, Fernandez MA, Dorph DJ, Betcher AM. Inguinal hernia repair. Comparison of local, epidural, and general anesthesia. New York state journal of medicine. 1979;79(11):1730-3.
2. Tverskoy M, Cozacov C, Ayache M, Bradley EL, Kissin I. Postoperative Pain After Inguinal Herniorrhaphy with Different Types of Anesthesia. Anesthesia & Analgesia. 1990;70(1):29-35.
3. Callesen T, Bech K, Kehlet H. One-thousand consecutive inguinal hernia repairs under unmonitored local anesthesia. Anesthesia and analgesia. 2001;93(6):1373-6, table of contents.
4. Sungurtekin H, Sungurtekin U, Erdem E. Local anesthesia and midazolam versus spinal anesthesia in ambulatory pilonidal surgery. Journal of Clinical Anesthesia. 2003;15(3):201-5.
5. Kehlet H, Aasvang E. Groin hernia repair: anesthesia. World journal of surgery. 2005;29(8):1058-61.
6. Chang FC, Farha GJ. Inguinal herniorrhaphy under local anesthesia. A prospective study of 100 consecutive patients with emphasis of perioperative morbidity and patient acceptance. Arch Surg. 1977;112(9):1069-71.
7. O'Dwyer PJ, Serpell MG, Millar K, Paterson C, Young D, Hair A, et al. Local or general anesthesia for open hernia repair: a randomized trial. Annals of surgery. 2003;237(4):574-9.
8. Kingsnorth AN, Britton BJ, Morris PJ. Recurrent inguinal hernia after local anaesthetic repair. The British journal of surgery. 1981;68(4):273-5.
9. Nordin P, Haapaniemi S, van der Linden W, Nilsson E. Choice of anesthesia and risk of reoperation for recurrence in groin hernia repair. Annals of surgery. 2004;240(1):187-92.

10. Zhang JM, An J. Cytokines, inflammation, and pain. International anesthesiology clinics. 2007;45(2):27-37.
11. Cruickshank AM, Fraser WD, Burns HJ, Van Damme J, Shenkin A. Response of serum interleukin-6 in patients undergoing elective surgery of varying severity. Clin Sci (Lond). 1990;79(2):161-5.
12. Kehlet H. Surgical stress response: does endoscopic surgery confer an advantage? World journal of surgery. 1999;23(8):801-7.
13. Di Vita G, Milano S, Frazzetta M, Patti R, Palazzolo V, Barbera C, et al. Tension-free hernia repair is associated with an increase in inflammatory response markers against the mesh. The American Journal of Surgery. 2000;180(3):203-7.
14. Hill ADK, Banwell PE, Darzi A, Menzies-Gow N, Monson JRT, Guillou PJ. Inflammatory markers following laparoscopic and open hernia repair. Surgical Endoscopy. 1995;9(6):695-8.
15. Gürleyik Md E, Gürleyik Md G, Çetinkaya Md F, Ünalmiser Md S. The Inflammatory Response to Open Tension-free Inguinal Hernioplasty Versus Conventional Repairs. The American Journal of Surgery. 1998;175(3):179-82.
16. Goldenberg A, Matone J, Marcondes W, Herbella FAM, Farah JFdM. Comparative study of inflammatory response and adhesions formation after fixation of different meshes for inguinal hernia repair in rabbits. Acta Cirurgica Brasileira. 2005;20: 347-52.
17. Schwab R, Eissele S, Brückner UB, Gebhard F, Becker HP. Systemic inflammatory response after endoscopic (TEP) vs Shouldice groin hernia repair. Hernia : the journal of hernias and abdominal wall surgery. 2004;8(3):226-32.
18. Carli F, Mayo N. Measuring the outcome of surgical procedures: what are the challenges? British journal of anaesthesia. 2001;87(4):531-3.
19. Cassuto J, Sinclair R, Bonderovic M. Anti-inflammatory properties of local anesthetics and their present and potential clinical implications. Acta anaesthesiologica Scandinavica. 2006;50(3):265-82.
20. Giddon DB, Lindhe J. In vivo quantitation of local anesthetic suppression of leukocyte adherence. The American journal of pathology. 1972;68(2):327-38.
21. MacGregor RR, Thorner RE, Wright DM. Lidocaine inhibits granulocyte adherence and prevents granulocyte delivery to inflammatory sites. Blood. 1980;56(2):203-9.
22. Schmidt W, Schmidt H, Bauer H, Gebhard MM, Martin E. Influence of lidocaine on endotoxin-induced leukocyte-endothelial cell adhesion and macromolecular leakage in vivo. Anesthesiology. 1997;87(3):617-24.

23. Azuma Y, Shinohara M, Wang PL, Suese Y, Yasuda H, Ohura K. Comparison of inhibitory effects of local anesthetics on immune functions of neutrophils. International journal of immunopharmacology. 2000;22(10):789-96.
24. Ohsaka A, Saionji K, Sato N, Igari J. Local anesthetic lidocaine inhibits the effect of granulocyte colony-stimulating factor on human neutrophil functions. Experimental hematology. 1994;22(5):460-6.
25. Lan W, Harmon DC, Wang JH, Shorten GD, Redmond PH. Activated endothelial interleukin-1beta, -6, and -8 concentrations and intercellular adhesion molecule-1 expression are attenuated by lidocaine. Anesthesia and analgesia. 2005;100(2): 409-12.
26. Hollmann MW, Gross A, Jelacin N, Durieux ME. Local anesthetic effects on priming and activation of human neutrophils. Anesthesiology. 2001;95(1):113-22.
27. Mikawa K, Akamarsu H, Nishina K, Shiga M, Obara H, Niwa Y. Effects of ropivacaine on human neutrophil function: comparison with bupivacaine and lidocaine. European journal of anaesthesiology. 2003;20(2):104-10.
28. Doran C, Yi X. The anti-inflammatory effect of local anesthetics. The Pain Clinic. 2007;19(5):207-13.
29. Amid PK, Shulman AG, Lichtenstein IL. Open "tension-free" repair of inguinal hernias: the Lichtenstein technique. The European journal of surgery = Acta chirurgica. 1996;162(6):447-53.
30. Amid PK, Shulman AG, Lichtenstein IL. Local anesthesia for inguinal hernia repair step-by-step procedure. Annals of surgery. 1994;220(6):735-7.

## Postoperative Clinical Outcomes and Inflammatory Markers after Inguinal Hernia Repair with Local or Spinal or General Anesthesia: A Randomized Trial

**Recruit patients**

**Inclusion criteria**

Patient ASA I–III, age ≥ 18 ปี undergoing inguinal hernia

**Exclusion criteria**

- allergy to any medication used this study
- femoral hernia
- recurrent or bilateral inguinal hernia
- bleeding abnormalities
- severe hepatic, renal or cardiovascular disease
- chronic use of opioid
- hx of using steroid/NSAIDs in the past 6 months
- inability to communicate

**Before operation: A blood sample 7 mL will be collected before prescribe iv fluid administration**

Randomly allocated

**Local anesthesia (LA)**

Sedation: Dormicum 1 mg and fentanyl 25 mcg

Patients will receive local anesthesia with 1% lidocaine adrenaline

**Spinal anesthesia (SA)**

Sedation: Dormicum 1 mg and fentanyl 25 mcg

0.5% heavy bupivacaine 15 mg will be injected to initiate sensory block at T4 level

**General anesthesia (GA)**

Induction: propofol, fentanyl

Airway: LMA

Maintain: Sevo in 60% O2, fentanyl (if need)

Before finish operation: 0.5% bupivacaine 10 ml will locally be infiltrated around incision.

**Postoperative pain control**

1. Oral acetaminophen one-two tablets every 6 hours
2. Arcoxia® (etoricoxib) 60-90 mg daily unless contraindicated
3. morphine 1-2 mg IV will be provided prn q 4 h as a breakthrough medication

POD 8 hr after operation: **Pain at rest and on mobilization (score 0-10) will be records**
**A blood sample 7 mL will be collected ->**

POD 24 hr after operation: **Pain at rest and on mobilization (score 0-10) will be records**
**A blood sample 7 mL will be collected -> send to department of Microbiology, Faculty of Medicine Siriraj Hospital. Tel. 0241970000 ext 8409 or contact Navin Tel. 0816490040**

**Total postop analgesics: Morphine-dose**
**Paracetamol, NSAIDs**

**Complication: N/V, urinary retention, bleeding/hematoma, fever, infection Patient satisfaction**

**In the LA group**,
patients will receive local anesthesia, 0.5% bupivacaine plus 2% lidocaine with adrenaline (1:200,000) (maximum dose is 300 mg in plain form, and 500 mg with epinephrine), similar to that described by Amid et al. Surgeons will be taught to do the local anesthetic technique in a standardized manner.

**In the SA group**,
patients will be positioned in the lateral position and a Whitacre 25 G needle will be inserted at L3-4 intervertebral space and then heavy bupivacaine 0.5% 15 mg will be injected. Sensory block (T4 and below dermatomes) to cold and pinprick will be tested before starting operation.

An incremental dose containing 1 mg of midazolam and 25 mcg of fentanyl will be intravenously given if patients in the LA and SA group require.

**In the GA group**,
patients will be induced with propofol 2 mg/kg and fentanyl 1.5 µg /kg. They are then allowed to breathe spontaneously with sevoflurane 2% to 2.5% in a mixture of 60% oxygen through a laryngeal mask. End-tidal concentration of sevoflurane will be adjusted to keep end-tidal sevoflurane 1MAC. Supplemental doses of 25 µg of fentanyl will be administered if intraoperative heart rate and blood pressure are greater than 20% of baseline.

**Postoperative analgesia**,
- All groups will receive infiltration 10 ml of 0.5% bupivacaine into the surgical wounds.
- Oral acetaminophen two tablets every 6 hours
- Arcoxia® (etoricoxib) 60-90 mg daily unless contraindicated for the duration of their hospital stay
- Intravenous morphine 1-2 mg will be provided every 4 hours as a breakthrough medication.

2 PRANNOK Rd. BANGKOKNOI
BANGKOK 10700

Tel. +66 2419 2667-72
Fax. +66 2411 0162

**Siriraj Institutional Review Board**

**Certificate of Approval**

**COA no. Si 157/2013**

**Protocol Title** : Postoperative Clinical Outcomes and Inflammatory Markers after Inguinal Hernia Repair with Local or Spinal or General Anesthesia: A randomized Trial

**Protocol number** : 035/2556(EC1)

**Principal Investigator/Affiliation** : Assist. Prof. Mingkwan Wongyingsinn, M.D. / Department of Anesthesiology
Faculty of Medicine Siriraj Hospital, Mahidol University

**Research site** : Faculty of Medicine Siriraj Hospital

**Approval includes :**

1. SIRB Submission Form
2. Proposal
3. Participation Information Sheet
4. Informed Consent Form
5. Case Record Form
6. Principle Investigator's curriculum vitae

**Approval date** : March 11, 2013

**Expired date** : March 10, 2014

This is to certify that Siriraj Institutional Review Board is in full Compliance with international guidelines for human research protection such as the Declaration of Helsinki, the Belmont Report, CIOMS Guidelines and the International Conference on Harmonization in Good Clinical Practice (ICH-GCP).

(Prof. Jarupim Soongswang, M.D.)
Chairperson

19 MAR 2013
date

(Clin. Prof. Udom Kachintorn, M.D.)
Dean of Faculty of Medicine Siriraj Hospital

21 MAR 2013
date



พิมพ์ที่หน่วยพิมพ์โรงพยาบาลศิริราช 2341 / 3,000 แผ่น / มิ.ย.55 / M / Mat.10023252

**All Siriraj Institutional Review Board Approved Investigators must comply with the Following :**

1. Conduct the research as required by the Protocol ;
2. Use only the Consent Form bearing the Siriraj Institutional Review Board "APPROVED" stamp ;
3. Report to Siriraj Institutional Review Board all of serious illness of any study subject ;
4. Promptly report to Siriraj Institutional Review Board any new information that may adversely affect the safety of the subjects or the conduct of the trial ;
5. Provide reports to Siriraj Institutional Review Board concerning the progress of the research, when requested ;
6. Conduct the informed consent process without coercion or undue influence, and provide the potential subject sufficient opportunity to consider whether or not to participate.
